# Automated closed-loop resuscitation of multiple hemorrhages: a comparison between fuzzy logic and decision table controllers in a sheep model

**DOI:** 10.1186/s40696-016-0029-0

**Published:** 2017-01-09

**Authors:** Nicole Ribeiro Marques, Brent J. Ford, Muzna N. Khan, Michael Kinsky, Donald J. Deyo, William J. Mileski, Hao Ying, George C. Kramer

**Affiliations:** 1 University of Texas Medical Branch, 301 University Boulevard, Galveston, TX 775555 USA; 2 Wayne State University, 45050 Anthony Wayne Drive, Detroit, MI 48202 USA

**Keywords:** Fluid resuscitation, Hypotension, Animal model, Traumatic brain injury, Fuzzy logic control, Decision table control

## Abstract

**Background:**

Hemorrhagic shock is the leading cause of trauma-related death in the military setting. Definitive surgical treatment of a combat casualty can be delayed and life-saving fluid resuscitation might be necessary in the field. Therefore, improved resuscitation strategies are critically needed for prolonged field and en route care. We developed an automated closed-loop control system capable of titrating fluid infusion to a target endpoint. We used the system to compare the performance of a decision table algorithm (DT) and a fuzzy logic controller (FL) to rescue and maintain the mean arterial pressure (MAP) at a target level during hemorrhages. Fuzzy logic empowered the control algorithm to emulate human expertise. We hypothesized that the FL controller would be more effective and more efficient than the DT algorithm by responding in a more rigid, structured way.

**Methods:**

Ten conscious sheep were submitted to a hemorrhagic protocol of 25 ml/kg over three separate bleeds. Automated resuscitation with lactated Ringer’s was initiated 30 min after the first hemorrhage started. The endpoint target was MAP. Group differences were assessed by two-tailed *t* test and alpha of 0.05.

**Results:**

Both groups maintained MAP at similar levels throughout the study. However, the DT group required significantly more fluid than the FL group, 1745 ± 552 ml (42 ± 11 ml/kg) versus 978 ± 397 ml (26 ± 11 ml/kg), respectively (*p* = 0.03).

**Conclusion:**

The FL controller was more efficient than the DT algorithm and may provide a means to reduce fluid loading. Effectiveness was not different between the two strategies. Automated closed-loop resuscitation can restore and maintain blood pressure in a multi-hemorrhage model of shock.

## Background

Hemorrhagic shock and traumatic brain injury (TBI) are major causes of death after traumatic injuries in the United States [1, 2]. In addition to hemostasis, the primary treatment of hypovolemic shock is to restore blood volume and end-organ oxygen delivery. Differential approaches for intravenous fluid therapy depend on the mechanism of injury [2, 3]. Fluid resuscitation efforts target a normal to low blood pressure after a severe hemorrhage. With traumatic brain injury (TBI), resuscitation efforts target a higher blood pressure because cerebral perfusion of the injured brain is pressure dependent. In both cases, initial therapy for casualty care would titrate fluid to the minimum volume necessary to achieve therapeutic goals based on the nature of the injury. This therapy would limit over-resuscitation, and provide logistic advantages by minimizing the field supplies needed for combat casualty care. Identification of a clinical physiological variable that closely reflects global or regional hypoperfusion or cellular hypoxia, and thus could be potentially used as best resuscitation target during shock remains to be defined [4]. Titration of fluid based on available vital signs is probably the most practical approach to being used on the battlefield and during transport.

Casualty transport to definitive care potentially requires multiple evacuations that often take hours. During transport, caregiver numbers are often limited and lack the ideal expertise to make decisions regarding adequate interventions. Automated closed-loop systems have the potential to enhance en route care, reducing caregiver time-effort and allowing the medic or corpsman to focus on other critical tasks and other casualties. Computerized systems allow individualized target setup and enable changes of endpoints during the treatment course. One attractive approach to closed-loop controllers is using fuzzy logic control. The primary thrust of the fuzzy logic control paradigm is to utilize human control operator’s knowledge and experience in the form of fuzzy sets, fuzzy “if-then” rules, and fuzzy reasoning to intuitively construct a controller able to emulate human behavior to a certain extent. Compared to the traditional control paradigm, the advantages of the fuzzy logic are two folds. First, a mathematical model of the system to be controlled is not required, and a satisfactory nonlinear controller can often be developed empirically in practice without complicated mathematics. Proper use of fuzzy logic control can significantly shorten product research and development time with reduced cost.

We reported previously on a closed-loop resuscitation system that used a simple decision table algorithm [5, 6]. In the present study, we compared the performance of a decision table algorithm and a fuzzy logic controller for fluid infusion control, to rescue and maintain mean arterial pressure (MAP) at a target level during hemorrhages. We assessed effectiveness, the ability to maintain MAP on target; and efficiency, the volume of fluid required to achieve target endpoints. We hypothesized that the fuzzy logic controller would be more effective and more efficient than the decision table algorithm.

## Methods

The experimental protocol was reviewed and approved by the Institutional Animal Care and Use Committee of the University of Texas Medical Branch at Galveston, with adherence to National Institutes of Health guidelines for care and use of laboratory animals.

### Animal preparation

Ten adult female Merino ewes (2–3 years old, 34–50 kg) were obtained from a U.S. Department of Agriculture licensed vendor (USDA license 74-B-0555; Talley Ranch, Uvalde, TX). The animals were held in quarantine for 15 days in 12-h light/dark cycles and examined by a veterinarian to confirm their health status. The animals were housed at a temperature of 23 °C in groups of two to four and had access to food and water ad libitum. The animals were fasted overnight and sedated with an intramuscular injection of ketamine (5 mg/kg), followed by general anesthesia induced and maintained with Isoflurane (2–5%). The animals were intubated and mechanically ventilated with a FiO_2_ of 0.5, tidal volume of 10 ml/kg, and respiratory rate of 10–15 breaths per minute to achieve an end-tidal CO_2_ of 35–40 mmHg. Pre- and postsurgical analgesia was provided with long-acting intramuscular buprenorphine (0.3 mg). The animals were surgically instrumented one week prior the study with two femoral arterial and two venous catheters. Arterial catheters were used for blood pressure monitoring, blood sampling, and hemorrhaging; venous catheters were used for fluid infusion. A transit time Doppler flow probe (20–24 mm A-probe, Transonic Systems, Ithaca, NY) was placed around the common pulmonary artery to measure continuous cardiac output (CO). Splenectomy was performed through a lateral flank approach to eliminate autotransfusion of splenic contraction during hemorrhage. After surgical preparation, the animals were monitored for a core temperature, blood cell count, discomfort, and pain.

Monitoring during the study was performed for arterial blood pressure, CO, and heart rate. Transducer outputs (Monitoring Kit with Flush Device, Abbott Industries, North Chicago, IL) were attached to a clinical monitor (Hewlett Packard, Model 78534C, Andover, MA) and calibrated. The analog signal was digitized (100 Hz) and recorded using an analog–digital converter (PowerLab, AD Instruments Inc., Colorado Springs, CO) on a Macintosh computer (Apple, Cupertino, CA). Blood samples were taken at the baseline period before the hemorrhage and throughout the study for monitoring blood gases and lactate (iStat Portable Clinical Analyser, iStat Corp, East Windsor, NJ). Oxygen delivery (DO_2_) was calculated as the product of the CO and the arterial oxygen content. The animals were catheterized with a 14 French foley catheter and urine output was collected via a Criticore Fluid Output Monitor (BARD, Covington, GA).

### Experimental protocol

**Fig. 1:**
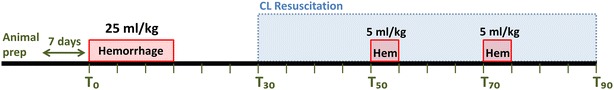
Study protocol. Animal surgical instrumentation (Animal prep), start of hemorrhage (T0). 30 min (T30), 50 min (T50), 70 min (T70), and 90 min (T90) after protocol began

The studies were performed on conscious sheep housed in a metallic cage. The protocol was previously described by Vaid [5] and Rafie [6]. In summary, the animals were hemorrhaged a total of 35 ml/kg over three separate bleeds after a baseline period. All events are referenced to the start of the first hemorrhage, designated time point 0 (T0), Fig. 1. The first bleed simulated a major hemorrhage of 25 ml/kg over 15 min (T0–T15). Closed-loop resuscitation was started at T30 and continued until T90. The second and third bleeds were 5 ml/kg over 5 min starting at T50 and T70, respectively.

The animals were divided into two treatment groups of five animals each: closed-loop controlled based on a decision table algorithm (DT group), and a fuzzy logic controller (FL group). The treatment goal was to restore and maintain MAP at 80 mmHg by controlling the infusion rate of lactated Ringer’s solution.

The animals were euthanized with intravenous ketamine (25 mg/kg) followed by saturated KCL (1 ml/kg) after completion of the experiments.

### Closed-loop system

Resuscitation was automated with a closed-loop control that incorporated three functional components: a physiologic sensor via the vital sign monitor (arterial pressure transducer), a computer, and an infusion pump. MAP and the algorithms were processed by LabView software (National Instruments Corp, Austin, TX), which controlled a commercial intravenous infusion pump (modified Power Infuser, Infusion Dynamics Inc., Plymouth Meeting, PA). A sampling of the MAP signal was at 100 Hz, while data was passed to the controller algorithm every 5 s for the FL group, and every 2 min for the DT group. The Power Infuser™ (ZOLL Power Infuser modified by Arcos, TX) is a small (320 g) battery operated intravenous pump developed for combat casualty care. The maximum infusion rate was set at a clinically realistic 100 ml/min per 70 kg (1.43 ml/min/kg) and was delivered when the MAP was lower than 40 mmHg.

Pilot studies were performed to tune the algorithms so that the controllers were able to reach and maintain MAP at a target level. The decision table algorithm was based on a lookup table with 5 ranges of blood pressure, each with a different infusion rate [5, 6]. The decision tablet algorithm was empirically designed to aggressively resuscitate when MAP was low, but as MAP approached target the infusion rate fall off to a very low flow. This was done to avoid over-shooting the desired MAP. The maximum flow was set when MAP was equal to or lower than 40 mmHg, 80% of the maximum infusion rate was set when MAP was between 41 and 44 mmHg, 60% of the maximum infusion rate was set when MAP was between 45 and 49 mmHg, 30% of the maximum infusion rate was set when MAP was between 50 and 69 mmHg, and 10% maximum infusion rate when MAP was between 70 and 89 mmHg. It is noted that 10% of the maximum infusion rate is 10 ml/min per 70 kg. At 90 mmHg and above, there was no infusion rate. It had previously been found that an effective means to maintain MAP at a target level using the decision table algorithm, was to continue to infuse a small volume at a low rate until 10 mmHg above target MAP [5, 6].

The fuzzy logic controller uses two linear input fuzzy sets for each of the two input signals, MAP error and rate change of MAP error, four Takagi–Sugeno fuzzy control rules with the simplified linear rule consequents, and the centroid defuzzifier. Ying [7, 8] showed that this fuzzy controller is a nonlinear proportional-integral controller with variable proportional-gain and integral-gain. The gains change with the input signals in a manner that enhances the control performance, resulting in better performance than a comparable linear proportional-integral controller, especially for nonlinear systems, or systems with large time delays, which was the case for our study. More detail of this controller is given in the “Appendix”.

All controllers were implemented in LabView software package running on a Macintosh desktop computer with a National Instruments I/O card (DaqCard 6024E PCMCIA).

### Statistics

No power calculation was performed a priori. The performance of each group was assessed by comparing the measured MAP to the target MAP. Adequate MAP control was defined as maintaining MAP in the range of ±5 mmHg of the target, expressed as a percentage of study time. Control performance parameters were assessed by applying control statistics to include performance error (PE) as the percent difference between measured and desired MAP, bias as median performance error (MDPE), and inaccuracy as median absolute performance error (MDAPE) [9]. Data is presented as mean ± standard deviation (SD), with group differences assessed by two-tailed *t* test and alpha of 0.05. Correction for multiple comparisons was performed with Holm-Sidak method. Data analysis was performed with GraphPad Prism 7.02 (GraphPad Software, San Diego-CA, USA).

## Results

**Fig. 2:**
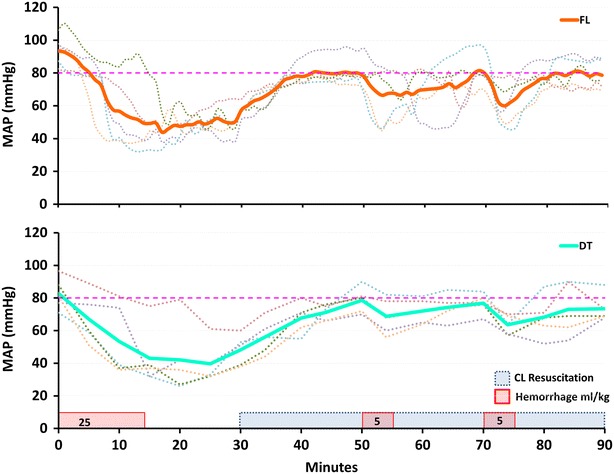
Average (*solid line)* and individual (*dotted lines*) mean arterial pressure (MAP) during experimental protocol for the fuzzy logic (FL) and decision table (DT) groups. Target MAP is marked as a *dashed line*. *CL* Closed-loop

All animals survived the hemorrhage protocol. The baseline MAP was not different between the FL and DT groups, (mean ± SD) 95 ± 9 and 83 ± 10 mmHg respectively (*p* = 0.1). The MAP responses during the first hemorrhage were similar between the groups, reaching a nadir of 35 ± 6 mmHg and 36 ± 14 mmHg for the FL and DT groups, respectively (*p* = 0.9). The MAP drop (ΔMAP) during the first hemorrhage was not different between the groups, 60 ± 9 mmHg for the FL and 47 ± 9 mmHg for the DT group, *p* = 0.06. The MAP in both groups showed partial recovery prior to the start of the closed-loop resuscitation, 51 ± 11 mmHg for the FL and 48 ± 9 mmHg for the DT group, *p* = 0.9. Figure 2 shows the MAP over time for both groups. The average result is plotted as a solid line, with each individual study plotted as a dotted line.

**Fig. 3:**
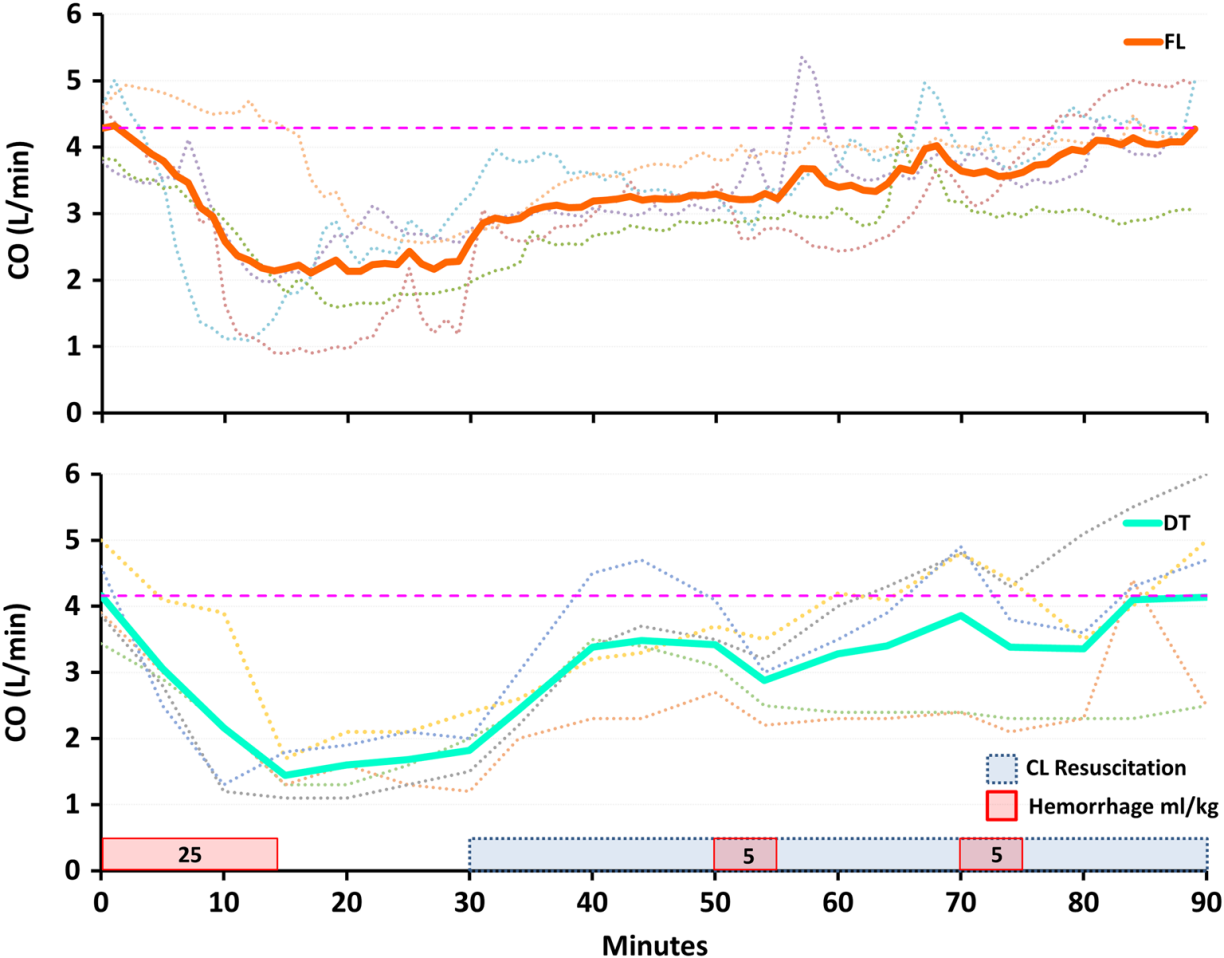
Average (*solid line*) and individual (*dotted lines*) cardiac output (CO) during experimental protocol for the fuzzy logic (FL) and decision table (DT) groups. Baseline CO is marked as a *dashed line*. *CL* Closed-loop

There were identical CO during the baseline period, 4.3 ± 0.5 l/min for the FL and 4.2 ± 0.6 l/min for the DT group (*p* = 0.9). During the first hemorrhage, the drop in CO from baseline was similar between the groups, reaching 2.3 ± 0.7 l/min for the FL and 1.3 ± 0.2 l/min for the DT group (*p* = 0.2). Both treatment groups were able to increase CO to baseline values at the end of the study protocol. Figure 3 shows CO during the experiment.

Arterial blood gas analysis including pH, pO_2_, and pCO_2_ were similar between the groups at baseline and throughout the study. The hemoglobin concentration trended higher in the DT group with a significant difference at baseline (*p* = 0.02). The baseline DO_2_ and its drop during the study were similar between the groups. The base deficit was significantly higher for the DT group at baseline and throughout the study. The lactate levels were significantly higher for the DT group at T30, T60 and T90 (*p* < 0.05). Results are reported in Table 1.

**Table 1:** Oxygen delivery (DO_2_), lactate and hemoglobin levels at the beginning of the protocol (T0), 30 (T30), 60 (T60) and 90 (T90) min

	Group	T0	T30	T60	T90
pH	FL	7.5 ± 0.1	7.5 ± 0.1	7.4 ± 0.1	7.5 ± 0.0
	DT	7.5 ± 0.1	7.4 ± 0.1	7.4 ± 0.0	7.4 ± 0.0
pO_2_ (mmHg)	FL	84 ± 6	94 ± 9	100 ± 22	104 ± 8
	DT	76 ± 9	79 ± 14	87 ± 20	87 ± 7
pCO_2_ (mmHg)	FL	35 ± 7	32 ± 10	35 ± 6	36 ± 9
	DT	31 ± 5	27 ± 5	25 ± 2	26 ± 1
Base excess (mmol/l)	FL	7.0 ± 2.4	−0.5 ± 3.1	−1.0 ± 1.5	1.2 ± 1.7
	DT	1.0 ± 3.2*	−6.8 ± 2.6*	−9.8 ± 3.3*	−9.4 ± 3.1*
DO_2_ (ml/min)	FL	410 ± 106	175 ± 38	198 ± 23	229 ± 74
	DT	468 ± 60	166 ± 44	227 ± 37	245 ± 55
Lactate (mmol/l)	FL	0.9 ± 0.3	5.2 ± 2.5	5.0 ± 2.5	5.3 ± 2.8
	DT	1.2 ± 0.6	9.6 ± 0.6*	11.0 ± 1.6*	11.5 ± 1.8*
Hemoglobin (g/dl)	FL	7.9 ± 1.2	6.5 ± 1.3	5.6 ± 1.4	4.8 ± 0.5
	DT	9.8 ± 0.8*	7.9 ± 0.5	6.0 ± 0.9	5.3 ± 1.0

Data is presented as mean ± standard deviation

*pO*_*2*_ Partial pressure of oxygen, *pCO*_*2*_ partial pressure of carbon dioxide, *DO*_*2*_ oxygen delivery, *FL* fuzzy logic group, *DT* decision table group

* *p* < 0.05

### Effectiveness

The DT group restored the MAP to the target level after the first and second hemorrhages, and the FL group reached target after all three hemorrhages. There were not significant MAP differences between the groups after each hemorrhage. During the resuscitation period, the percentage of time that the controllers were able to maintain the MAP between 75–85 mmHg was 27 ± 18% for the FL group, and 23 ± 21% for the DT group (*p* = 0.9). The percentage of time that MAP was between 70–90 mmHg was 51 ± 21% for the FL group, and 49 ± 32% for the DT group (*p* = 0.9). Resuscitation was able to maintain CO above 80% of the baseline value for 39 ± 40% of the time for the FL group, and 43 ± 31% of the time for the DT group (*p* = 0.8).

The performance statistics showed no difference between the groups. The PE was −8.5 ± 4.7% for the FL group, and −14.2 ± 8.4 for the DT group (*p* = 0.5). Bias calculated as MDPE was −5.6 ± 5.5% for the FL group, and −11.2 ± 10.3% for the DT group (*p* = 0.5). Inaccuracy calculated as MDAPE was 10.3 ± 4.5% for the FL group, and 14.2 ± 6.4 for the DT group (*p* = 0.5).

**Fig. 4:**
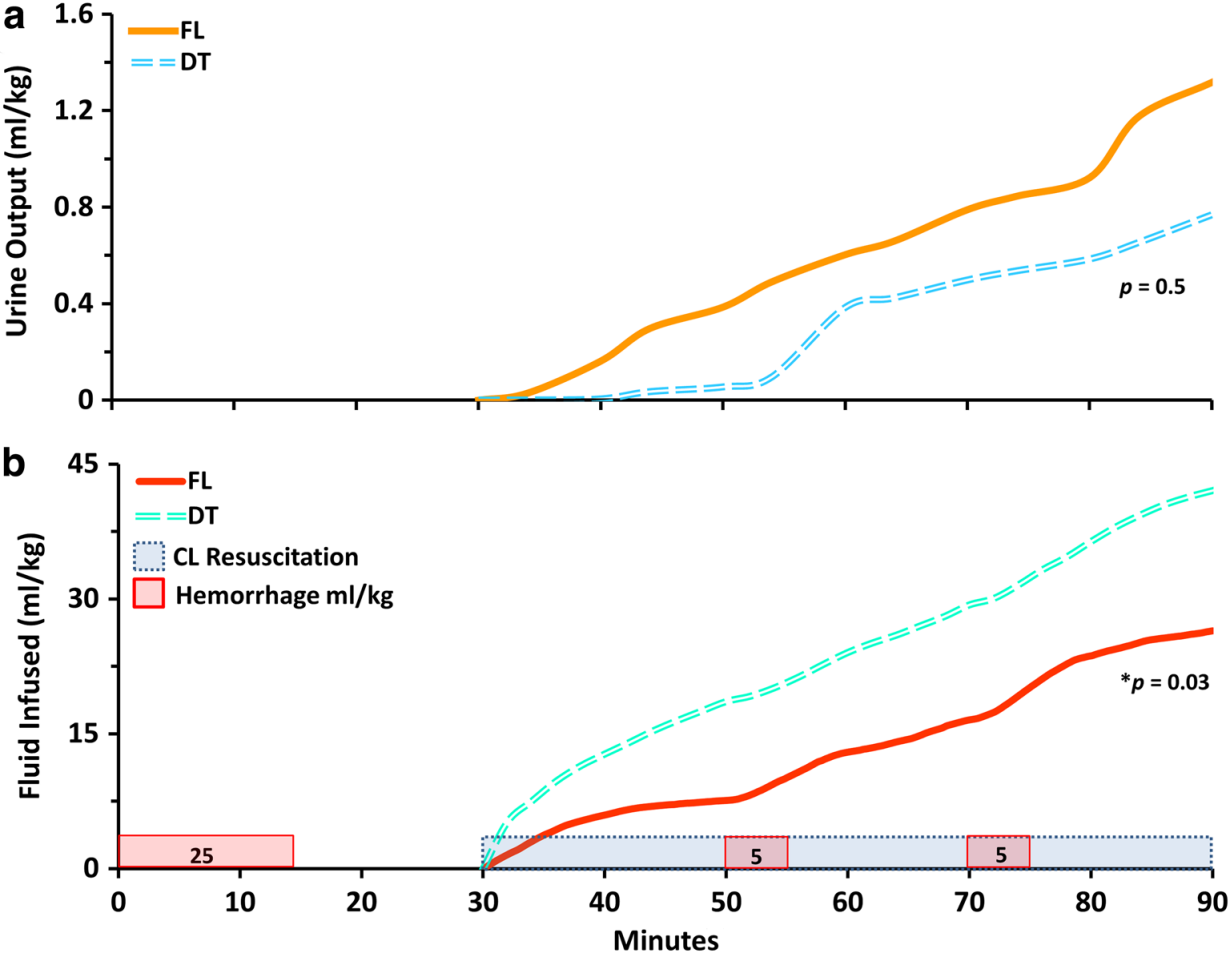
**a** Urine output over time for the fuzzy logic (FL) and decision table (DT) groups. **b** Cumulative fluid infused over time for the fuzzy logic (FL) and decision table (DT) groups. *CL* Closed-loop

During the 60 min resuscitation period, all animals in the FL group had urine output equal to or greater than 0.5 ml/kg/h, while 2 animals in the DT group had urine output level of 0.02 and 0.3 ml/kg/h. There was no statistical difference in urine output between the groups during resuscitation (Fig. 4a).

### Efficiency

Figure 4b depicts the total volume of lactated Ringer’s infused for each group: 978 ± 397 ml (26 ± 11 ml/kg) for the FL group, and 1745 ± 552 ml (42 ± 11 ml/kg) for the DT group (*p* = 0.03). The average ratio of the volume infused per volume bled was 0.8 and 1.2 for the FL and DT groups, respectively. The average infusion rate was significantly higher for the DT group compared to the FL group, 28 ± 9 versus 16 ± 7 ml/min, respectively (*p* = 0.04).

## Discussion

In the present study, we tested a closed-loop resuscitation system and compared the performance of two algorithms to deliver fluids using blood pressure as a primary feedback. A controlled hemorrhage model was chosen as it allows for an identical and reproducible model of hypovolemia.

Our first hypothesis is rejected as both algorithms were equally effective and showed no performance difference. Even with intermittent hemorrhages, the FL and DT groups were able to reach the target and maintain MAP within 5 mmHg of the target level for 27 and 23% of the resuscitation time, respectively The CO of both groups presented a drop greater than 50% from the baseline after the first hemorrhage, and were restored to baseline values towards the end of the study.

The DT group required significantly more fluid than the FL group to maintain similar MAP levels (42 vs. 26 ml/kg, *p* = 0.03). The data supports our second hypothesis, the fuzzy logic controller was more efficient than the decision table algorithm. These results can be considered surprising showing an uncoupling of the resuscitation volumes infused, and its impact on blood pressure.

The average hemoglobin in the DT group was significantly higher at baseline, but no difference was evident after the first hemorrhage. Oxygen delivery capacity was severely impaired after the first hemorrhage on both groups, and a small recovery of oxygen supply was seen during fluid resuscitation. However, DO_2_ were not different between the two treatments throughout the study. Further, the DT group had a significantly greater base deficit and higher levels of lactate at T30, T60, and T90 (Table 1). Low DO_2_ and high lactate levels could be considered resuscitation triggers for most providers and further lifesaving interventions including blood products and surgery would need to be considered.

The FL group accounted for a greater challenge compared to the DT group considering a significantly lower hemoglobin prior resuscitation. Despite this, the FL controller was able to keep hemodynamic variables similarly to the DT group and infused less fluid volume. The ratio of volume infused per volume bled was low for both groups (0.8 for the FL and 1.2 for the DT), despite the normotensive target. Both groups were able to provide resuscitation titrated to blood pressure with close to zero net fluid balance. There was no significant difference in urine output between the groups however it trended higher in the FL group (Fig. 4a). Closed-loop systems may provide a means to reduce fluid excess and extend the time before transfusion.

Although blood pressure does not accurately predict global tissue perfusion, it correlates well with coronary and cerebral blood flow [10, 11], and so is a practical surrogate for perfusion. On the battlefield, polytrauma patients with major hemorrhages requiring urgent field resuscitation will most likely have only traditional vital signs available to guide initial care. Fluid resuscitation strategies must infuse the minimal amount necessary to avoid complication from fluid overload e.g. dilution of clotting factors, and profound hypoperfusion e.g. acute kidney injury. Over- and under-resuscitation of a combat casualty may be avoided by targeting the resuscitation efforts to patient needs, mechanism of injury, goals set by the caregiver and adherence to guidelines. Autonomous systems continuously assess patient’s status, can rapidly identify hemodynamic decompensation, and automatically implement therapy or alert caregivers when intervention is necessary. Autonomous systems have shown to be superior to manual infusion when fine control is needed [12–14].

Closed-loop resuscitation may also provide a real-time index of changes in physiologic status. Fluid infusion rates increase during hemorrhage and decrease after hypotension has been corrected. Thus, increases in the resuscitation effort or infusion rate may reflect an unrecognized internal bleed that could alter triage or provide an earlier identification of a non-responder. Increased infusion rates are visible in Fig. 4 during the second and the third hemorrhages (line slopes).

Civilian trauma differs from a military combat setting, especially in regards to evacuation times, safe resuscitation zones, personnel expertise, and supplies. In the urban environment the approach relies on the short transport time of a civilian casualty to a trauma center with definitive care implemented by a trauma team; in this case the primary goal of prehospital care is to transport the casualties as rapidly as possible, and not to attempt major interventions at the scene [15]. In a military setting, though, evacuation times can be longer, reported to be on average 90 min between 2001 and 2009, and estimated to reach up to 72 h [16]. When definitive surgical treatment of a combat casualty is delayed, life-saving fluid resuscitation is often necessary in the field. Therefore, enhanced resuscitation strategies are critically needed for prolonged field and en route care. We suggest that automated resuscitation systems are a potential means to improve fluid resuscitation, cope with inter-individual variability, and allow simultaneous care of multiple casualties.

### Limitations

This study has several limitations. Damage control resuscitation is a strategy of delivering blood as initial therapy and may be the preferred fluid infused in future conflicts. While there is an associated risk of developing metabolic derangements and coagulopathy with crystalloid resuscitation, the survival benefit outweighs this risk in critically injured patients and can be a lifesaving intervention [17–20]. Crystalloid (isotonic solutions) is the fluid of choice for the treatment of acute hypovolemic shock according to the Advanced Trauma Life Support (ATLS) book [21]. Crystalloid was used as the first step in the evaluation of the algorithm and further studies should include blood products.

The coagulation profile was not assessed during this study protocol, therefore it is unknown the extent of the crystalloid resuscitation on a likely acute traumatic coagulopathy. However, the lower volume administered in the FL group suggest less dilution of the coagulation factors. Our study protocol simulated the first 90 min of a hemorrhagic injury, to include the golden hour of treatment of traumatic injuries. The goal was not to complete resuscitation, but rather enable the casualty to reach definitive care where blood products and a surgical team would provide the next level of lifesaving interventions.

While a widely-held view is that blood pressure is not the best variable to assess the level of hypovolemia; treatment of hemorrhagic shock continues to use blood pressure as a primary endpoint. In particular trauma with TBI requires prompt treatment and prevention of hypotension, as a single episode of hypotension can double mortality risk [22]. Invasive and noninvasive measurements e.g., CO and DO_2_, and specific derived variables e.g., compensatory reserve index [24] have been suggested as better resuscitation endpoints. However, none of these are proven practical for early care of combat casualties and have failed to show a clear benefit in terms of patient outcomes [23].

We studied a rapidly responding closed-loop system with arterial blood pressure measured continuously to allow prompt adjustment of fluid infusion rates. However, on the battlefield only standard noninvasive vital signs are available. This limitation may be overcome in the future as several continuous noninvasive blood pressure monitors are being introduced to the marketplace [25–27]. An intermittent cuff pressure may provide sufficient data for a system that adjusts fluids every few minutes. Further, during en route care for combat casualties, noninvasive continuous blood pressure is likely to be available and provided by technologies such as the Office of Naval Research’s automated critical care system (ACCS) [28].

## Conclusion

Our experiment design replicates an initial and early treatment of multiple hemorrhages. Our findings suggest that hemodynamic derangement triggered by profound hemorrhagic shock can be corrected with fluid titration strategies using MAP as a target endpoint. Overall, our preliminary results suggest that fuzzy logic-based automated closed-loop fluid resuscitation can restore and maintain blood pressure in a multi-hemorrhage model and provide a means to effectively treat hemorrhages with less fluid volumes. Future research on the clinical effectiveness and safety of closed-loop fluid therapy is warranted.

## Figures and Tables

**Fig. 5:**
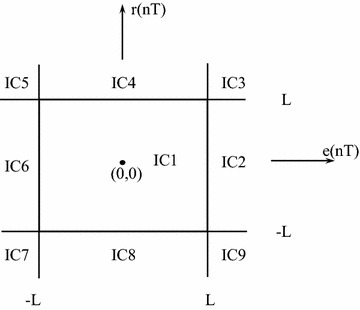
Nine ICs to cover all possible combinations of the fuzzy controller’s input signals, *e*(*nT*) and *r*(*nT*)

## Appendix: The fuzzy controller designed for this study

The fuzzy controller whose configuration was outlined in the main body was developed by Ying. His analysis showed that this fuzzy controller was actually a nonlinear proportional integral (PI) controller with variable proportional-gain and integral-gain [7].

Let *MAP*(*nT*) designate MAP value at sampling time *nT*, where *T* = 5 s was the sampling period in this study and *n* = 0,1,…. The following filter was used:

$$MAP_{f} (nT) = \frac{1}{4}\left( {MAP(nT) + MAP(nT - T) + MAP(nT - 2T) + MAP(nT - 3T)} \right)$$

With the initial condition being *MAP*(−*T*) + *MAP*(−2*T*) + *MAP*(− 3*T*) = *MAP*(0).

When *MAP*_*f*_(*nT*) < 40, the fuzzy controller output *U*(*nT*) = *U*_max_, which was the maximum fluid volume. *U*_max_ = Animal body weight (kg) × 1.429 in unit of ml/h. Whenever *MAP*_*f*_(*nT*) ≥ 40, the fuzzy controller calculated *Δu*(*nT*) as follows

$$\Delta u(nT) = \beta \left( {2.85e(nT) + 0.58r(nT)} \right)$$

where $e(nT) = {\text{setpoint}} - MAP_{f} (nT)$; *r*(*nT*) = *MAP*_*f*_(*nT* − *T*) − *MAP*_*f*_(*nT*). Which were the two input signals to the fuzzy controller. Here, the set point was the target MAP desired by the user. The value of *β* was calculated using the formulas in Table 2 according to the values of *e*(*nT*) and *r*(*nT*) that had nine possible combinations as shown in Fig. 5. These formulas were the exact representations of the fuzzy controller. They were derived from this specific fuzzy controller configuration [7]. For this study, we experimentally determined that the following parameter values for Table 2 produced the best control performance: *L* = 16, *k*_1_ = 1, *k*_2_ = 0, *k*_3_ = 0, *k*_4_ = 0.33.

**Table 2:** Formulas for computing value of ß in (1)

IC No.	β
1	Expression (1) below
2	k_1_[(1-k_2_)r(nT) + (1 + k_2_)L]/2L
3	k_1_
4	k_1_[(1-k_3_)e(nT) + (1 + k_3_)L]/2L
5	k_1_k_3_
6	k_1_[(k_3_-k_4_)r(nT) + (k_3_ + k_4_)L]/2L
7	k_1_k_4_
8	k_1_[(k_2_-k_4_)e(nT) + (k_2_ + k_4_)L]/2L
9	k_1_k_2_

Which formula to use depends on location of the controller’s two input signals (Fig. 5)

During the operation, after computing *Δu*(*nT*), the fuzzy controller then calculated the following:

$$u(nT) = U(nT - T) + \Delta u(nT)$$

where the initial condition *U*(−*T*) = 0. Then it computed the final output of the controller (i.e., the input signal to the fluid pump):

$$U(nT) = \left\{ {\begin{array}{*{20}ll} {u(nT)}, & {{\text{if}}\;0 \le u(nT) \le U_{\text{max} } } \\ {U_{\hbox{max} },} \hfill &\quad {{\text{if}}\;u(nT) > U_{\text{max} } } \\ {0,} & {{\text{if}}\;u(nT) < 0} \\ \end{array} } \right.$$

1 $$\beta = \frac{k_{1} }{4L^{2} }\left[ \left( 1 + k_{2} + k_{3} + k_{4} \right)L^{2} + (1 + k_{2} - k_{3} - k_{4} )L \cdot e(nT) + (1 - k_{2} + k_{3} - k_{4} )L \cdot r(nT){ + }(1 - k_{2} - k_{3} + k_{4} )e(nT)r(nT) \right]$$

## Abbreviations

DT: decision table
FL: fuzzy logic
MAP: mean arterial pressure
TBI: traumatic brain injury
USDA: United States Department of Agriculture
FiO_2_: fraction of inspired O_2_
CO: cardiac output
DO_2_: oxygen delivery
pO_2_: partial pressure of oxygen
pCO_2_: partial pressure of carbon dioxide
T0: time point zero (start time of the first hemorrhage)
PE: performance error
MDPE: median performance error
MDAPE: median absolute performance error
ATLS: advanced trauma life support
ACCS: automated critical care system
